# Role of Cyclin-Dependent Kinase Inhibitors in Endometrial Cancer

**DOI:** 10.3390/ijms20092353

**Published:** 2019-05-12

**Authors:** Gaia Giannone, Valentina Tuninetti, Eleonora Ghisoni, Sofia Genta, Giulia Scotto, Gloria Mittica, Giorgio Valabrega

**Affiliations:** 1 Department of Oncology, University of Turin, Via Giuseppe Verdi, 8, 10124 Turin, Italy; gaia.giannone@ircc.it (G.G.); valentina.tuninetti@ircc.it (V.T.); eleonora.ghisoni@ircc.it (E.G.); sofia.genta@ircc.it (S.G.); giulia.scotto@ircc.it (G.S.); 2 Candiolo Cancer Institute-FPO-IRCCS, Strada Provinciale 142 km 3.95, 10060 Candiolo, Turin, Italy; 3 Units of Oncology, ASL Verbano Cusio Ossola (VCO), Via Fiume, 18, 28922 Verbania, Italy; gloria.mittica@aslvco.it

**Keywords:** cyclin-dependent kinase inhibitors, endometrial cancer, cyclin-dependent kinases

## Abstract

Endometrial Cancer (EC) is an important cause of death in women worldwide. Despite early diagnosis and optimal treatment of localized disease, relapsed patients have few therapeutic options because after first line therapy, currently no standard of care exists. On the basis of endocrine positivity of most endometrioid ECs, Endocrine Therapy (ET) is a reasonable and widely accepted option. Better knowledge of molecular mechanisms involved in cancer highlighted the deregulated activity of Cyclin-Dependent Kinases (CDKs) in the cell cycle as a hallmark of carcinogenesis supporting the development of a new class of drugs: CDK inhibitors (CDKis). The aim of this review is to give an overview on CDKis preclinical, early clinical activity and future development in EC. Use of CDKis has a strong preclinical rationale but we have poor clinical data. Similar to breast cancer, most ongoing trials are investigating synergistic associations between CDKis and ET. These trials will probably help in defining the best clinical setting of CDKis in ECs, which are the best partner drugs, and how to manage CDKis toxicities with a focus on potential biomarkers of response.

## 1. Introduction

In 2018, endometrial cancers (ECs) accounted for about 63.230 new cases and 11.350 deaths in women in the United States [1]. The median age of diagnosis is 62 years, and risk factors are related to hyperestrogenism (i.e., early menarche, late menopause, polycystic ovary syndrome (PCOS), infertility but also obesity and diabetes) [2,3]. Indeed, Estrogens stimulate growing and proliferation of the endometrium during the menstrual cycle and are involved in development of EC [4]. Genetic predisposition plays a role in younger patients, being the incidence of Lynch Syndrome in up to 9% in patients with diagnosis of EC before the age of 50 [5].

EC is usually diagnosed at an early-stage, with vaginal bleeding being the most frequent clinical manifestation [2]. Patients with early-stage disease, on the basis of risk of relapse, are treated with surgery alone [6] followed by brachitherapy (BT) [7] or external beam radiation therapy (EBRT) [8] with a 5-year survival rate of more than 90%. Patients with locally advanced disease could be treated with a combination of surgery, chemotherapy and radiation therapy [9,10]. Patients with distant metastasis at initial diagnosis account for less than 10% but in this setting, prognosis remains poor, with a 5-year survival rate of less than 20% [11]. The standard of care is represented by systemic therapy with carboplatin-paclitaxel and palliative radiotherapy [12]. There is no standard of care in subsequent lines, but Endocrine Therapy (ET) is considered an option [13,14]. Indeed, more than 80% of EC specimens show Estrogen Receptor (ER) and Progesterone Receptor (PgR) positivity [15] and estrogens play a key role in EC development and progression [4]. ET is characterized by a manageable toxicity profile but its activity in EC has not been certainly defined [12]. As a matter of fact, in spite of similarities between EC and Breast Cancer (BC), ET does not play a main role in EC and nowadays there is no evidence that ET prolongs Overall Survival (OS) or Disease Free Survival (DFS) in this malignancy [12,13]. On the other hand, a meta-analysis focusing on Hormone receptors (HRs) evaluated the clinico-patological features that could predict sensitivity to ET [16]. The work showed that patients treated with ET have a 20.4% Overall Response Rate (ORR) with a median Progression Free Survival (PFS) of 2.8 months and a median OS of 10.2 months. Patients with ER or PgR positivity had higher ORR compared with ER or PgR negative patients [17]. This work has several limitations and lack of other evidence suggests the necessity to go beyond the mere HR expression to find biomarkers of response. 

Several endocrine therapies have been developed, among which progestins, Selective ER Modulators (SERMs), Selective ER Degraders (SERDs) and Aromatase Inhibitors (AIs) are used in clinical practice [18,19].

As underlined before, prognosis of advanced ECs is still poor. Treatment of advanced, relapsed and metastatic patients is still an unmet clinical need, and the role of ET in ECs has to be elucidated. Moreover, EC patients usually have several comorbidities, being obesity and diabetes important risk factors [20]. All these issues complicate therapeutic strategies. A better understanding of the molecular mechanism and a translational approach to these malignancies led to development of new compounds [21]. Interesting strategies are targeting the Vascular Endothelial Growth Factor (VEGF) pathway [22], using epigenetic drugs [21] or immunotherapy above all in “inflamed ECs” [23]. The PI3K/AKT/mTOR pathway, being downstream HRs, has been exploited with drugs like everolimus in combination with AIs [24]; on the other hand, drugs that inhibit Insulin Growth Factor Receptors (IGFRs), like metformin, are combined with AIs with or without PI3K-inhibitors, on the basis of IGFRs’ activity in the proliferation of the endometrium [21]. The main goal of these drugs is to enhance ET activity, without adding unacceptable toxicities to a well-tolerated regimen. Unfortunately, none of these compounds has currently changed the natural history of EC [21]. In this context, the development of drugs acting on the cell cycle in synergy with endocrine therapy, like Cyclin-Dependent Kinase inhibitors (CDKis), may represent a significant step forward in EC treatment. 

The aim of this review is to describe the rationale behind CDKis development, CDKis data of activity in ECs and future perspectives for development of these drugs. In this review, we highlight EC molecular and pathological classifications that play a role in defining prognosis but will probably also drive therapeutic choices in the near future (for example, suggesting which ECs could benefit from Immunotherapy), focusing on frequently altered pathways which may be relevant for CDKis treatment. Moreover, we discuss the general state of the art on CDKis, their preclinical activity and future perspective in ECs. 

## 2. Endometrial Cancer: Molecular Classification of Subtypes

EC has been historically classified into type I and type II on the basis of clinical characteristics and histological features [25,26]. Type I EC accounts for 60–70% of all ECs. It is characterized by grade 1 or 2 tumour differentiation with endometrioid histology, ERs and PgRs positivity. It is associated with endometrial hyperplasia and is related to obesity, hyperlipemia and hyperestrogenism (late onset of menopause, estrogen exposure). It has good prognosis with 5 year-OS of 86% [25,26]. The most frequently altered pathways are the PI3K/AKT/mTOR pathway, with PTEN mutation in more than half of samples [27], and the WNT signalling pathway (2–45%) [26,27].

Type II EC accounts for about 30% of all ECs. It is predominantly serous, clear cell or grade 3 endometrioid, with low or absence of ERs and PgRs. It is associated with endometrial atrophy and has poor prognosis with 5-year OS of 59% [25]. Type II EC is characterized by TP53 mutation (more than 60% of samples), PIK3CA mutation and HER2 amplification [26,27]. 

This model has some limits, because it is difficult for some tumours to be histologically classified, and some mutations recur in both types of EC. This classification has been recently enriched and will probably be replaced by The Cancer Genome Atlas (TCGA). 

It identifies 4 subgroups on the basis of copy number alteration, mutations and Microsatellite Instability (MSI): POLE-ultra-mutate, MSI-hyper-mutated (MSI-H), copy-number low and copy-number high subgroup [23,28]:

1. POLE-ultramutated malignancies, accounting for 17.4% of high-grade endometrioid tumours, are characterized by a high mutational burden with somatic mutations in the exonuclease domain of POLE which encodes the catalytic subunit of polymerase epsilon. Despite the histological grade, this group is associated with good prognosis [29,30,31].
2. MSI-hypermutated tumours represent 28.6% of low-grade and 54.3% of high-grade endometrioid EC. They are characterized by MSI and a high mutation rate; they are related to alteration of Mismatch Repair (MMR) system (the most commonly implicated genes are MLH1, MSH2, MSH6, PMS2) [23,28,32].
3. Copy number low subgroup is typically composed by low-grade endometrioid tumours with low mutation rate and microsatellite stability (MSS). PTEN and PIK3 are mutated in 77% and 90% samples, respectively. This subgroup is characterized by high expression of ER and PgR [23,28].
4. Copy number-high serous-like tumours have serous or mixed histology. They have a low mutation rate and a small load of copy number aberrations. TP53 is commonly mutated (91.7%). They show HER2-amplification and deregulation of the cell cycle (in particular with CCNE1 overexpression [17]). Prognosis of these patients is poor [23,28].

## 3. Cell Cycle Checkpoints and Cyclin Dependent Kinases Complexes 

The cell cycle is the set of phases in which DesoxyriboNucleicAcid (DNA) and cellular components double and divide into daughter cells [33]. The cell cycle consists of four phases called G1 (Gap 1), S (synthesis), G2, M (mitosis) [34]. In G1-phase, the cell grows and synthesizes all cellular components that are essential for DNA duplication. During S-phase, DNA is duplicated with high fidelity machinery [35]. In G2-phase, the cell prepares for the division that occurs in the next M-phase. Mitosis is the final phase during which two identical daughter cells are generated [34].

The cell cycle is carefully regulated in each phase by cyclins, CDKs, and CDKis [34,36]. CDKs are serine/threonine protein kinases that form active complexes with cyclins. These complexes are essential to drive cell cycle progression and transition into different phases [34,35].

At present we know that the CDK family is composed of 20 molecules [37], while 11 molecules are classified in the cyclin family [36]. These enzymes play a main role in three checkpoints that regulate cell cycle transition: G1 checkpoint, G2 checkpoint and M checkpoint [33]. All these checkpoints have been largely studied and the essential activity of these complexes has been elucidated. G1/S phase transition is ruled by CDK4/6-cyclin D and CDK2-cyclin E complexes. In response to mitogenic and growth signals, during G1 phase, CDK4/6 and cyclin D form a complex that phosphorylates retinoblastoma susceptible protein (Rb) causing the dissociation of E2F factor from Rb [38]. The role of E2F is to induce S-phase progression in association with the CDK2-cyclin E complex [34]. Moreover, during this phase, cyclin A removes cyclin E and forms a new complex, CDK2-cyclin A. During G2-phase, CDK2-cyclin A and CDK1-cyclin B complexes phosphorylate Forkhead box protein M1 (FoxM1) and activate the expression of FoxM1 target genes that codify for the transition to M-phase [36]. CDK1 activity is controlled by the balance between WEE kinases (WEE1 and myelin transcription factor 1 (MYT1)) and Cdc25 phosphatases. WEE1 is a tyrosine kinase that phosphorylates CDK1 at Tyr 15, and MYT1 is dual-specific for phosphorylation at Thr 14 and Tyr 15. These two kinases are responsible for inhibiting CDK1 phosphorylation. Cdc25 activates CDK1 by removing phosphates from residues and cells can enter M-phase. Also, CDK7 has an important regulatory role across different cell-cycle phases. CDK7 phosphorylates and activates other CDKs, thus acting as a CDK-activating kinase (CAK) [39]. This kinase complex consists of three subunits: the regulatory subunit cyclin H, the catalytic subunit CDK7 and a third protein factor, named Menage a trois 1 (Mat1). CAK activates CDK1, CDK2, CDK4 and CDK6 by threonine phosphorylation, regulating cell cycle progression [40].

Lastly, M checkpoint is where the attachment of the spindle fibers to centromeres is assessed. Only if this is correct mitosis will proceed [35]. All these checkpoints and kinases guarantee an efficient cell replication and preserve DNA integrity. Indeed, until DNA damages are repaired, the cell cycle is stopped and checkpoints prevent cells from going on to the next phase.

**Figure 1:**
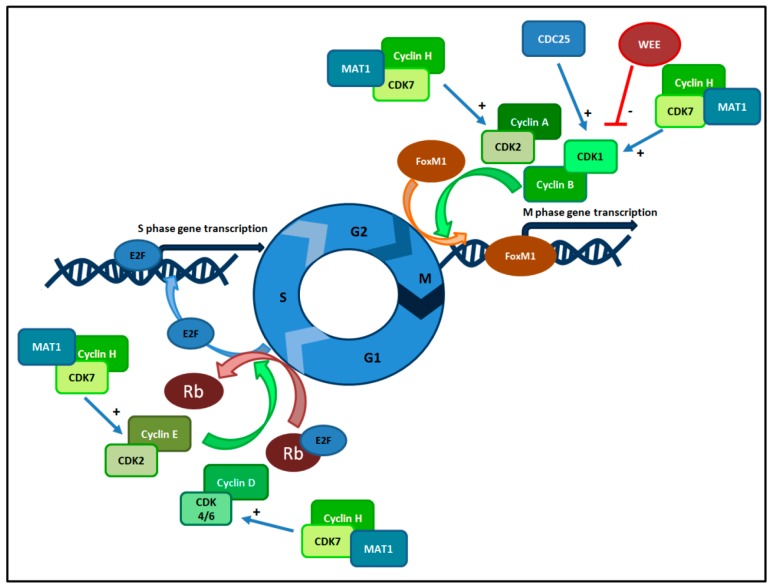
Cell Cycle and CDK complexes.

For an overview of the cell cycle and the most important Cyclin-CDK complexes, see Figure 1.

The cell cycle consists of four phases (G1, S, G2, M) regulated by cyclins, Cdks, and Cdkis. G1/S phase transition is controlled by Cdk4/6-cyclin D and Cdk2-cyclin E complexes. During G1 phase, Cdk4/6 and cyclin D form a complex that phosphorylates Rb causing the dissociation of E2F factor from Rb, thus inducing DNA transcription and S phase progression in association with the CDK2-cyclin E complex. During S phase, DNA is duplicated. During G2-phase, Cdk2-cyclin A and Cdk1-cyclin B complexes activate the expression of FoxM1 target genes that are essential for transition to M-phase. Cdk1 activity is controlled by the balance between WEE and Cdc25 phosphatases. Also, Cdk7 has an important regulatory role across different cell-cycle phases. Cdk7 phosphorylates and activates other Cdks, in combination with Mat1 and Cyclin H.

## 4. CDKis: Generations and Features

Four generations of CDKis have been currently developed [41]:

1. First generation (e.g., alvocidib): characterized by low potency (pan-CDK), lack of specificity and off target toxic effects;
2. Second generation (e.g., dinaciclib, CYC065): characterized by a non-selective inhibition of a wide range of CDKs [42] and equivalent activity in normal and tumour cells. Among these drugs, Dinaciclib inhibits Myc and shows interesting activity in triple negative BC cell lines, synergizing with PARP inhibitor niraparib and increasing DNA damage [43];
3. Third generation (e.g., palbociclib, ribociclib and abemaciclib): characterized by selectivity for both a subset of CDKs and tumor cells compared to untransformed cells, as reported below;
4. Fourth generation (e.g., ON-123300, TG02): characterized by higher potency than first generation and by a broad spectrum of activity against other pathways such as angiogenesis. These compounds are under investigation in preclinical and clinical settings.

First generation CDKis, in particular alvociclib, are characterized by an important cytotoxic effect in vitro and in vivo, both in tumour and normal cells, with acute tumour lyses as the main adverse event (AE) in chronic lymphocytic leukemia (CLL) [44]. Myelosuppression and diarrhea are the main grade 3 and 4 (G3–G4) toxicities reported in phase I studies [44,45,46], with 100% of G4 neutropenia and 75% of G3 diarrhea in cohorts receiving dose escalation. 

In second generation CDKis, the most common G3 and G4 AEs registered in patients treated with dinaciclib [47] were neutropenia (35.0%, with febrile neutropenia in 10.0% of patients), thrombocytopenia (20.0%), pneumonia and sepsis (5.0% each) with half of the patients having a serious AE in dinaciclib cohort. Third generation CDKis have a more manageable safety profile. The most frequent G3–G4 AE described with palbociclib and ribociclib is neutropenia. However, neutropenia associated with these two drugs is different from chemotherapy-induced neutropenia because it is quickly reversible, reflecting a cytostatic effect on neutrophil precursors [48]. Hematologic AEs are less common with abemaciclib, while fatigue and gastrointestinal toxicity are predominant; in particular, diarrhea was seen in more than 90% of patients (any grade) in MONARCH-1 and it was manageable with conventional antidiarrheal agents or dose reduction [49].

For a summary of CDKis generations and features see Table 1.

## 5. CDKis Development and Approval 

The setting in which CDKis have been clinically developed is HR-positive BC. In HR-positive BC, ER activates the promoter of CCND1, a gene encoding for cyclin D. Indeed, cyclin D is amplified or overexpressed in 20% and 50% of cases, respectively, while mutations of RB are rare [50,51,52]. On the other hand, cyclin D1 binds to ER and enhances its transcriptional activity [50] suggesting that crosstalk between the cyclin D pathway and ER pathway could be therapeutically exploited. On the contrary, triple negative BC is characterized by frequent RB mutation and overexpression of cyclin E [53,54].

Indeed ER-positive cell lines were more sensitive to palbociclib than Triple negative BC [55]. Moreover, ET and CDKis synergize in vitro [56,57]; this synergy is probably related to simultaneous inhibition of cyclin D by ET and CDK4/6 by CDKis. Lastly, ER positive BCs that develop endocrine resistance usually remain dependent on the cyclin D pathway, being inhibited in vitro by CDKis [58].

Third generation CDKis (palbociclib, ribociclib and abemaciclib) have been tested in large phase III trials in BC patients and have been recently approved in clinical practice [59].

PALBOCICLIB: Palbociclib is a selective inhibitor of CDK4 and CDK6 [60]. The chemical name of palbociclib is 6-acetyl-8-cyclopentyl-5-methyl-2-pyrido[2,3-d]pyrimidin-7(8H)-one [61]. It has been approved in combination with an AI for the treatment of HR-positive, HER2-negative advanced BC as a first line ET in postmenopausal women or in combination with fulvestrant for the treatment of HR-positive, HER2-negative advanced or metastatic BC in women with disease progression following endocrine therapy, on the basis of the PALOMA 2 study [62] and PALOMA 3 study [63], respectively.

ABEMACICLIB: Abemaciclib is a selective inhibitor of the complexes CDK4/cyclin D1 and CDK6/cyclin D1 [64]. The chemical name is N-(5-((4-ethylpiperazin-1-yl)methyl)pyridin-2-yl)-5-fluoro-4-(4-fluoro-1-isopropyl-2-methyl-1H-benzo[d]imidazol-6-yl)pyrimidin-2-amine. This compound acts as competitive inhibitor of the ATP-binding domain of CDK4 and CDK6 and seems to be more potent against CDK4 than against CDK6 [65]. To date, FDA approved abemaciclib in different settings:

1. In combination with fulvestrant, for the treatment of HR-positive, HER2-negative advanced or metastatic BC that progressed after ET on the basis of MONARCH 2 [66];
2. As monotherapy for patients with HR-positive, HER2-negative advanced or metastatic BC that progressed after ET and previous chemotherapy;
3. In combination with an AI as first-line ET for postmenopausal women with HR-positive HER2-negative advanced or metastatic BC on the basis of MONARCH 3 [67].

RIBOCICLIB: Ribociclib is a small molecule that selectively inhibits CDK4 and CDK6 [68]. The chemical name is 7-cyclopentyl-N,N-dimethyl-2-((5-(piperazin-1-yl)pyridin-2-yl)amino)-7H-pyrrolo[2,3-d]pyrimidine-6-carboxamide. FDA approved ribociclib in combination with an AI as initial endocrine-based therapy for the treatment of postmenopausal women (in 2017) and pre/perimenopausal woman with HR-positive HER2-negative advanced or metastatic BC (in 2018) according to results of the two phase 3 trials, MONALEESA-2 [68] and MONALEESA-7, respectively [69].

## 6. Predictors of Response and Resistance to CDKis

Nowadays, we have no predictors of response to CDKis in BC with the exception of ER positivity [70]. Indeed, studies focused on amplification of cyclin D, overexpression of CDK6 and expression of p16, a CDK inhibitor, as markers of response, but results were not definitive and further investigations are needed [70,71]. Moreover, cyclin E was deemed as a marker of acquired resistance to inhibitors of CDK 4/6 but evidence is weak [72]. Expression of Rb protein and the level of its phosphorylation seemed to predict response to CDKis, but in contrast, Rb nuclear expression was not associated with response to palbociclib in a phase II trial suggesting the need for additional studies [71]. An exploratory analysis of PALOMA-3 focused on acquired resistance, using circulating tumour DNA (ctDNA) to define if there are common pathway of resistance to CDKis. It suggested that mutation of RB is a driver mutation in the palbociclib plus fulvestrant arm while mutations of PI3KCA (gene encoding for PI3K) and ESR1 (gene encoding for ER1) are driver mutations in both arms (fulvestrant alone and fulvestrant plus palbociclib), playing a main role in acquired endocrine resistance [63]. An important effort in defining mechanism of resistance is a study by Wander and colleagues [73]. They performed a Whole exome sequencing (WES) in 51 metastatic BC specimens of patients before treatment with CDKis and of 11 paired metastatic BC specimens after progression during CDKis treatment. This study suggests that several potential mechanisms could be involved in primary or acquired resistance to CDKis. Among these, RB1 inactivation, AKT1 mutation or amplification, Aurora Kinase A (AURKA) amplification seem to be the most frequent [73]. These results need to be confirmed by additional studies but could pave the way for more rational combination therapies in the near future. In conclusion, on the basis of interdependence of CDKis and the ER pathway, HR-positivity seems to be the only clinical predictor to identify patients that could benefit from the CDK4/6 inhibitor nowadays [70].

## 7. Preclinical Activity of CDKis in Endometrial Cancer

The cell cycle and its checkpoints play an important role in endometrial proliferation and cancer. Indeed, a high expression of cyclin A, cyclin D and cyclin E is frequent in EC. Moreover, estrogens and progesterone show opposite activity in the cell cycle as described below. This explains the rationale for the development of CDKis above all in type 1 EC.

EC shares with BC several features, suggesting that CDKis could be exploited in this malignancy. Indeed, EC expresses HR; ET is a therapeutic option and it shows a deregulation of the CDK pathway [74]. Cyclin A is usually expressed in EC and preclinical data suggest that high expression of cyclin A is associated with less differentiated tumours and shorter OS [75]. Moreover, EC is characterized by frequent cyclin D1 aberrations (16% of samples with 3′Untranslated Region (UTR) mutations that stabilize CCND1 messenger RiboNucleic Acid (mRNA); coding for frame mutations or amplifications) [65]. These alterations lead to cyclin D activating features that are predictive of response to Abemaciclib in preclinical models [65]. Also, Palbociclib shows preclinical activity in EC with RB expression (almost 70% of 337 specimens collected by Tanaka and colleagues), suppressing tumour growth, also significantly reducing ki67 and phosphor-RB expression in PDXs [76]. Palbociclib was also studied in PTEN (Phosphatase and tensin homolog) knockout mouse models with EC. This treatment significantly increased mouse survival, reducing tumour growth [77]. Also, combination of ET and CDK4/6 inhibitors seems to be an interesting option in type I EC. Indeed, preclinical studies suggest that Estradiol and Progesterone have opposite activity in terms of nuclear levels of p27, an inhibitor of CDK2-cyclin E and CDK4-cyclin D and consequently on cell cycle arrest in G1 phase. Estradiol induces p27 phosphorilation and subsequent p27 ubiquitylation and degradation, while Progesterone increases nuclear p27, inducing cell cycle arrest [78]. Moreover, Estradiol stimulated-endometrium shows high levels of cyclin D1 mRNA, therefore suggesting that activation of cyclin D1 plays a key role downstream of the ER pathway [79,80].

On the other hand, serous carcinoma of the uterus (USC) is an interesting setting for CDKis. It accounts for only 10% of all endometrial cancers but has a poor prognosis and few therapeutic options [81]. Almost 85% of these tumours harbor an altered expression of cell-cycle-related genes, with amplification of CCNE1 in 48% of cases [17,26,28]. Moreover, overexpression of cyclin E is associated with less differentiated tumours and low ER and PgR expression [82], similar to triple negative BC. Cocco and colleagues demonstrated that CYC065 (a second-generation, ATP competitive inhibitor of CDK2/9) is active in CCNE1-overexpressing USC cells. CYC065 reduces tumour growth in PDXs. It also synergizes with Taselisib (a new PI3K-inhibitor) in vitro and in PDX with CCNE1-amplified/PI3K-mutated USCs [83], suggesting a crosstalk of these two pathways.

## 8. Current Development of CDKis in Endometrial Cancer

ECs patients were enrolled in phase I dose escalation studies of the main CDK 4/6 inhibitors, reporting a manageable safety profile [84,85].

Palbociclib toxicity profile was evaluated in a phase I trial that enrolled 41 patients. Dose Limiting Toxicity (DLTs) was neutropenia (12%) with Maximum tolerated Dose (MTD) and Recommended Phase II Dose (RP2D) of 125 mg once daily. Among patients evaluated for tumour response, 27% had stable disease with 6 patients receiving at least 10 cycles [84].

Infante and colleagues evaluated the safety of ribociclib in 132 patients with different solid tumours and lymphomas expressing RB. MTD was established at 900 mg/day while RP2D was 600 mg/day in a 3 weeks on/one week off schedule. The most frequent G3-G4 Adverse Events (AEs) were neutropenia (27%), leukopenia (17%), while asymptomatic corrected QT prolongation was specific to doses ≥600 mg/day (9% of patients at 600 mg/day; 33% at doses >600 mg/day) [85]. Initial data of activity were collected with 3 partial responses and 43 patients with stable disease. Exploratory analysis with Next Generation Sequencing (NGS) suggests that alteration in CCND1 could predict ribociclib clinical activity. Indeed, a higher percentage of patients who were treated for ≥8 weeks had tumours with alterations in *CCND1* compared with patients who received ribociclib for <8 weeks and three of the four patients (75%) who had the longest clinical benefit were cyclin D amplified [85].

Abemaciclib shows a manageable profile of toxicity in a phase I trial with expansion cohorts that enrolled 225 patients. The only DLT was fatigue [86]. The MTD was 200 mg twice a day. The most common AE was diarrhea (all above grades 1 and 2) while the most frequent G3-G4 AE was neutropenia (10%). In an ER + BC expansion cohort, the Clinical Benefit Rate (CBR, including Complete Response, Partial Response and Stable Disease for more than 24 weeks) was 49%, with 61% of patients that received abemaciclib for more than 6 months [86].

As for other CDKis, seliciclib, an orally bioavailable inhibitor of several complexes (CDK2-cyclin E, CDK1-cyclin B, CDK7-cyclin H and CDK9-cyclin T1), has been evaluated in a phase I trial [87]. MTD was 800 mg twice a day for 7 days in a 21 day cycle, with anorexia, hypokalaemia, rash, rinse of creatinine and fatigue as the main DLTs. Concerns about toxicities stopped further development of this schedule and this dosage. On the other hand, preliminary results of the NCT00999401 trial suggest that a combination of seliciclib and sapacitabine is safe, being the most common G3-G4 AEs elevations in ALT (10%), AST (13%) and neutropenia (21%) [88].

Ongoing clinical trials are evaluating CDKis activity in ECs. Exploiting the crosstalk between the two pathways downstream, HR and CDKis in EC, CDKis can be used to potentiate activity of ET. Indeed, combinations of AI with one of three main CDK4/6 inhibitors are under development (NCT03643510, NCT02657928, NCT02730429) in phase II trials. NCT03643510 is evaluating activity of fulvestrant plus abemaciclib in metastatic EC patients that could have received only one prior line of ET and prior chemotherapy. NCT02657928 recruits both ECs and Ovarian Cancer patients receiving ribociclib and letrozole. A translational explorative analysis of this study is searching potential predictive biomarkers and is evaluating response in PDXs. NCT02730429 is a double blind placebo controlled phase II trial that evaluates activity of letrozole with or without palbociclib in patients that could have received no more than one prior ET. Interestingly, an inclusion criterion is that the tumour must be ER positive (with immunohistochemistry ≥10%). On the other hand, even more powerful combinations are under study, for example, the triplet of CDKis, ET and PI3K-inhibitors (NCT03008408, NCT03675893). These combinations are also under development in BC. Indeed, preclinical evidence suggests that endocrine resistance is mediated, at least in part, by interactions of ER with CDK4, resulting in PI3K hyperactivation [89] and that CDKis could overcome it [90]. Monitoring toxicities of these combinations will be crucial.

Lastly, despite interesting preclinical activity of CDKis in cyclin E amplified USC, nowadays we have no ongoing study in this specific setting.

For a list of the main ongoing clinical trials developing CDKis in ECs, see Table 2.

## 9. Discussion

Metastatic or recurrent ECs are still an important cause of morbidity and death in women worldwide and despite important efforts and new drugs, median OS has not significantly changed over the last years. In this context, better knowledge of molecular alterations led us understand that cell cycle checkpoint deregulation plays an important role in carcinogenesis and could represent an interesting strategy in different settings. On this basis, new-generation CDKis, with a specific target and a more manageable toxicity profile, have demonstrated outstanding activity in BC (PALOMA 2 and 3, MONARCH 3, MONALEESA 2 and 7), prolonging PFS in HR-positive BC patients. This paved the way for the employment of this class of drugs in other malignancies.

However, several questions are still open:

1. What role will CDKis play in ECs?
2. What are the best partner drugs?
3. How can we select patients that could benefit from CDKis?
4. Are toxicities manageable in these patients?
5. In EC, ET is an option in pretreated patients, above all in Type I EC that is characterized by strong HR-positivity, being a safe treatment in a setting of poor therapeutic options and in patients that usually have several comorbidities [16]. Nevertheless, clinical activity of ET is not fully elucidated and meta-analysis concluded that it does not improve OS, and we have insufficient data on its activity in reducing symptoms or improving Quality of Life [13]. Several efforts were done to optimize ET. In this setting, using deregulated pathways to enhance ET activity is an interesting option. Combinations with epigenetic modulators and drugs inhibiting the PI3K/AKT pathway or IGFR pathway are under development, but drugs that act on cell cycle checkpoints seem to be the most attractive strategy [21].
6. Although different combinations are under development, doublet with an AI and a CDKi has the strongest preclinical rationale on the basis of cyclin D1 activity, which facilitates ER transcriptional activity, inducing ER-related gene expression. On the other hand, activation of cyclin D1 is downstream of the ER pathway [79,80]. Moreover, ECs express Rb in 70% of cases with frequent mutations or amplification of cyclin D [65,76]. In this context, combination of AIs with CDKis will use crosstalk and alteration of these two pathways to enhance activity of ET. This has been clinically demonstrated in BC and will probably play an important role in type I EC on the basis of its endocrine dependency; three phase II trial are developing this combination (NCT02730429, NCT02657928, NCT03134638). Other combinations are under development and BC is paving the way. Ongoing studies are combining CDKis with AI and IGFR inhibitors (NCT03099174) or different PI3K/AKT inhibitors, acting on pathways that are deregulated in ECs. Lastly, combinations of CDKis and immunotherapy are under development on the basis of CDKis preclinical activity in the tumour microenvironment (TME). Indeed, they induce a higher expression of PD-1, enhance antigen presentation and reduce proliferation of immunosuppressive T regulators. Ongoing clinical trials are evaluating these combinations in different settings including MSI-High solid tumours (NCT02791334), suggesting CDKis and immunotherapy could be a powerful combination in MSI-Hypermutated ECs [91,92].
7. As described before, nowadays we have no clear predictor of response to CDKis with the exception of ER positivity in BC. Unfortunately, in ECs, ERs and PgRs, although having a role as prognostic factors, are not clear predictors of response to ET [21,93,94]. Moreover, response to ET in ECs is lower than in BCs, suggesting that drawing inference from BCs is not suitable for EC. Preclinical works suggest that different isoforms of ER and PgR could be related to different endocrine sensitivity [21], while mutations in ESR1, encoding for truncated forms of ER, recur in 20% of TGCA specimens [95]. Also, alteration of downstream pathways like the PI3K/AKT pathway [27], could play a role in endocrine resistance but further prospective studies are needed. In this context, some ongoing trials are selecting patients on the basis of ER positivity while others are conducting exploratory analysis to find predictors of response in EC. Lastly, BC development of CDKis is using window of opportunity trials in a neoadjuvant setting (i.e., NCT02441946). This could be applied to EC patients in the near future. Indeed a chemo-naïve population, receiving CDKis for a short period of time with the possibility of a tumour specimen after treatment (at time of surgery), could be the ideal setting to evaluate potential biomarkers of response in an homogeneous population.
8. Dose limiting toxicities have been the major concern of first and second generation CDKis. A growing experience in management of CDK4/6 inhibitors in BC patients demonstrates that these are long-term well tolerated drugs in this setting with transient hematologic toxicities or diarrhea that recover with dose reduction [48]. Unfortunately, we have few data on long-term toxicities of CDK4/6 inhibitors and on adverse events that could occur with newest combinations and in EC populations. Indeed, one of the major concerns in the development of new drugs in ECs is toxicity of these compounds in a frail population. As described before, these patients have comorbidities like diabetes and hypertension and in this context hyperglycemia and hypercolesterolemia, which are PI3K inhibitors common AEs [24] or proteinuria and hypertension that are VEGF inhibitors common AEs could become unacceptable toxicities [96]. On the other hand, strategies intend to improve ET activity but not at the cost of a higher percentage of AEs, being one of the major pros of ET its manageable profile in these patents. Common toxicities of CDKis are haematological or gastrointestinal, suggesting they could be tolerated in EC patients, but ongoing studies will elucidate this aspect.

## 10. Conclusions

Use of CDKis in ECs has a strong preclinical rationale and preclinical data on their activity are interesting. Moreover, EC intrinsic endocrine sensitivity, and its frequent deregulation of cell cycle suggest that CDKis could play a role in these patients, enhancing activity of ET. On the other hand, we have few clinical data, and no phase III trial is ongoing in this setting. Ongoing studies are evaluating the activity of CDKis in EC, focusing above all on combinations with AIs. Optimizing ET without adding unacceptable toxicities is the main goal of future CDKis development, but several questions are still open. Firstly, although ET and CDKis seem to be the most interesting combination, preclinical data on CDKis in CCNE1-expressing USC and on CDKis activity on TME suggest that CDKis development could be extended to MSI-Hypermutated EC in combination with immunotherapy and USC, but clinical trials are scarce. Secondly, we have few studies evaluating biomarkers of activity in EC and development of CDKis could be an opportunity to evaluate biomarkers of response in homogeneous populations. Thirdly, toxicities of CDKis in BC suggest that they have manageable side effects in patients that have several comorbidities, but further data are needed for EC patients.

In conclusion, ongoing trials will probably define the best settings and partner drugs of CDKis and will clarify AEs of CDKis in EC patients and how to manage these AEs.

## Figures and Tables

**Table 1:** CDKis generations and features.

	First Generation (e.g., Alvocidib)	Second Generation (e.g., Dinaciclib, CYC065)	Third Generation (e.g., Abemaciclib, Ribociclib, Palbociclib)	Fourth Generation (e.g., TG02)
Mode of action	Multi-serine/threonine CDKis	Inhibitor of a wide range of CDKs	Inhibitor of CDK4 and CDK6	Pyrimidine-based multi-kinase inhibitor (together with JAK2 and FLT3)
Side effects	-Tumour lyses syndrome (in CCL, reversible) -Myelosuppression -Diarrhea	-Neutropenia -Thrombocytopenia -Pneumonia -Sepsis	-Neutropenia -Fatigue -GE toxicity (diarrhea)	-early clinical development, side effects not yet available
Advantages	-First to demonstrate clinical activity in vitro	-activity shown in Multiple Myeloma -Enhance PARPis activity in vitro (Dinaciclib)	-Higher potency -Selective activity intumour cells -Cross BBB (abemaciclib) -Manageable safety profile -oral administration -administered once daily (palbociclib and ribociclib)	-Higher potency (non pan-CDK) -Large spectrum of activity (e.g., also angiogenesis) -Potent anti-proliferative effects in tumour cell lines
Limitations	-Low potency (pan-CDK) -Lack of specificity -Off target toxic effects -intravenous administration	-Non selective activity -Equivalent activity on normal and tumour cells -intravenous administration	-Prolonged QT interval (mandatory ECG before and during treatment with ribociclib)	-Under investigation in preclinical and clinical setting -dose-dependent

Legend: CDK: cyclin-dependent kinase; CCL: chronic lymphocytic leukemia; AML: acute myeloid leukemia; GE: gastro-enteric; BBB: blood brain barrier; JAK2: Janus Kinase 2; FLT3: fms-like tyrosine kinase-3; PARPi: PARP inhibitors.

**Table 2:** Main ongoing clinical trials with CDKis in Endometrial Cancer.

Description	Condition	Line of Therapy	Primary Endpoint	Phase	Status	Trial Identifier
**Palbociclib**						
Palbociclib	Ovarian teratomas, GCTs or tumours with alteration at the G1/S checkpoint.	na	ORR, Safety	II	Recruiting	NCT01037790
Palbociclib+ cisplatin or carboplatin	Solid tumours	na	%AEs, DLT, RP2D	I	Recruiting	NCT02897375
Palbociclib	Solid tumours with alteration at the G1/S checkpoint.	>2 line	ORR	II	Recruiting	NCT02465060
Letrozole+ Palbociclib/Letrozole +placebo	Metastatic EC	No more than 1 prior line of ET	PFS	II	Recruiting	NCT02730429
Palbociclib + Gedatolisib	Solid tumours	na	%AEs, DLT, RP2D	I	Recruiting	NCT03065062
**RIBOCICLIB**						
Ribociclib +Letrozole	OC and EC	na	%PFS at 12 weeks	II	Active, not recruiting	NCT02657928
Ribociclib+ everolimus + Letrozole	EC	≤ 3 line	DLT/CBR	I/II	Recruiting	NCT03008408
**ABEMACICLIB**						
LY3023414 (PI3Ki) and Abemaciclib +/− Letrozole	EC	na	PFS at 6 months, ORR	II	Not yet recruiting	NCT03675893
Fulvestrant+ Abemaciclib	EC	1 or 2 line	ORR	II	Recruiting	NCT03643510
**OTHER CDKIS**						
Dinaciclib + Veliparib	Solid tumours with BRCA mutation	na	RP2D	I	Recruiting	NCT01434316
Seliciclib+ Sapacitabine	Solid tumours	na	MTD	I	Unknown	NCT00999401
CYC065	Solid tumours or lymphomas	na	DLTs	I	Recruiting	NCT02552953

Legend: AEs: Adverse Events; DLTs: Dose Limiting Toxicities; EC: Endometrial Cancer; GCT: Granulosa Cell Tumours; MTD: Maximum Tolerated Dose; NA: not available; OC: Ovarian Cancer; ORR: Overall Response Rate; PI3Ki: PI3K inhibitor; RP2D: Recommended Phase II Dose.

## References

[1] Siegel R.L., Miller K.D., Jemal A. (2018). Cancer statistics, 2018. CA Cancer J. Clin..

[2] Braun M.M., Overbeek-Wager E.A., Grumbo R.J. (2016). Diagnosis and Management of Endometrial Cancer. Am. Fam Physician.

[3] McAlpine J.N., Temkin S.M., Mackay H.J. (2016). Endometrial cancer: Not your grandmother’s cancer. Cancer.

[4] Key T.J., Pike M.C. (1988). The dose-effect relationship between ‘unopposed’ oestrogens and endometrial mitotic rate: Its central role in explaining and predicting endometrial cancer risk. Br. J. Cancer.

[5] Lu K.H., Schorge J.O., Rodabaugh K.J., Daniels M.S., Sun C.C., Soliman P.T., White K.G., Luthra R., Gershenson D.M., Broaddus R.R. (2007). Prospective determination of prevalence of lynch syndrome in young women with endometrial cancer. J. Clin. Oncol..

[6] Signorelli M., Lissoni A.A., Cormio G., Katsaros D., Pellegrino A., Selvaggi L., Ghezzi F., Scambia G., Zola P., Grassi R. (2009). Modified radical hysterectomy versus extrafascial hysterectomy in the treatment of stage I endometrial cancer: Results from the ILIADE randomized study. Ann. Surg. Oncol..

[7] Nout R.A., Smit V.T., Putter H., Jürgenliemk-Schulz I.M., Jobsen J.J., Lutgens L.C., van der Steen-Banasik E.M., Mens J.W., Slot A., Kroese M.C. (2010). Vaginal brachytherapy versus pelvic external beam radiotherapy for patients with endometrial cancer of high-intermediate risk (PORTEC-2): An open-label, non-inferiority, randomised trial. Lancet.

[8] de Boer S.M., Powell M.E., Mileshkin L., Katsaros D., Bessette P., Haie-Meder C., Ottevanger P.B., Ledermann J.A., Khaw P., Colombo A. (2018). Adjuvant chemoradiotherapy versus radiotherapy alone for women with high-risk endometrial cancer (PORTEC-3): Final results of an international, open-label, multicentre, randomised, phase 3 trial. Lancet Oncol..

[9] Secord A.A., Geller M.A., Broadwater G., Holloway R., Shuler K., Dao N.Y., Gehrig P.A., O’Malley D.M., Finkler N., Havrilesky L.J. (2013). A multicenter evaluation of adjuvant therapy in women with optimally resected stage IIIC endometrial cancer. Gynecol. Oncol..

[10] Lee L.J., Viswanathan A.N. (2012). Combined chemotherapy and radiation improves survival for node-positive endometrial cancer. Gynecol. Oncol..

[11] Dowdy S.C. (2014). Improving oncologic outcomes for women with endometrial cancer: Realigning our sights. Gynecol. Oncol..

[12] Colombo N., Creutzberg C., Amant F., Bosse T., González-Martín A., Ledermann J., Marth C., Nout R., Querleu D., Mirza M.R. (2016). ESMO-ESGO-ESTRO Consensus Conference on Endometrial Cancer: Diagnosis, treatment and follow-up. Ann. Oncol..

[13] Kokka F., Brockbank E., Oram D., Gallagher C., Bryant A. (2010). Hormonal therapy in advanced or recurrent endometrial cancer. Cochrane Database Syst. Rev..

[14] Fleming G.F. (2015). Second-Line Therapy for Endometrial Cancer: The Need for Better Options. J. Clin. Oncol.

[15] Huijgens A.N., Mertens H.J. (2013). Factors predicting recurrent endometrial cancer. Facts Views Vis. Obgyn.

[16] Ethier J.L., Desautels D.N., Amir E., MacKay H. (2017). Is hormonal therapy effective in advanced endometrial cancer? A systematic review and meta-analysis. Gynecol Oncol.

[17] Zhao S., Choi M., Overton J.D., Bellone S., Roque D.M., Cocco E., Guzzo F., English D.P., Varughese J., Gasparrini S. (2013). Landscape of somatic single-nucleotide and copy-number mutations in uterine serous carcinoma. Proc. Natl Acad Sci USA.

[18] Thangavelu A., Hewitt M.J., Quinton N.D., Duffy S.R. (2013). Neoadjuvant treatment of endometrial cancer using anastrozole: A randomised pilot study. Gynecol. Oncol..

[19] Berstein L., Maximov S., Gershfeld E., Meshkova I., Gamajunova V., Tsyrlina E., Larionov A., Kovalevskij A., Vasilyev D. (2002). Neoadjuvant therapy of endometrial cancer with the aromatase inhibitor letrozole: Endocrine and clinical effects. Eur J. Obstet Gynecol. Reprod Biol..

[20] Arnold M., Pandeya N., Byrnes G., Renehan P.A.G., Stevens G.A., Ezzati P.M., Ferlay J., Miranda J.J., Romieu I., Dikshit R. (2015). Global burden of cancer attributable to high body-mass index in 2012: A population-based study. Lancet Oncol..

[21] Jerzak K.J., Duska L., MacKay H.J. (2019). Endocrine therapy in endometrial cancer: An old dog with new tricks. Gynecol. Oncol..

[22] Aghajanian C., Filiaci V., Dizon D.S., Carlson J.W., Powell M.A., Secord A.A., Tewari K.S., Bender D.P., O’Malley D.M., Stuckey A. (2018). A phase II study of frontline paclitaxel/carboplatin/bevacizumab, paclitaxel/carboplatin/temsirolimus, or ixabepilone/carboplatin/bevacizumab in advanced/recurrent endometrial cancer. Gynecol. Oncol..

[23] Mittica G., Ghisoni E., Giannone G., Aglietta M., Genta S., Valabrega G. (2017). Checkpoint inhibitors in endometrial cancer: Preclinical rationale and clinical activity. Oncotarget.

[24] Slomovitz B.M., Jiang Y., Yates M.S., Soliman P.T., Johnston T., Nowakowski M., Levenback C., Zhang Q., Ring K., Munsell M.F. (2015). Phase II study of everolimus and letrozole in patients with recurrent endometrial carcinoma. J. Clin. Oncol..

[25] Bokhman J.V. (1983). Two pathogenetic types of endometrial carcinoma. Gynecol. Oncol..

[26] Murali R., Soslow R.A., Weigelt B. (2014). Classification of endometrial carcinoma: More than two types. Lancet Oncol.

[27] Le Gallo M., Bell D.W. (2014). The emerging genomic landscape of endometrial cancer. Clin. Chem..

[28] Kandoth C., Schultz N., Cherniack A.D., Akbani R., Liu Y., Shen H., Robertson A.G., Pashtan I., Shen R., Benz C.C. (2013). Integrated genomic characterization of endometrial carcinoma. Nature.

[29] Stelloo E., Bosse T., Nout R.A., MacKay H.J., Church D.N., Nijman H.W., Leary A., Edmondson R.J., Powell M.E., Crosbie E.J. (2015). Refining prognosis and identifying targetable pathways for high-risk endometrial cancer; a TransPORTEC initiative. Mod. Pathol..

[30] Church D.N., Stelloo E., Nout R.A., Valtcheva N., Depreeuw J., ter Haar N., Noske A., Amant F., Tomlinson I.P., Wild P.J. (2015). Prognostic significance of POLE proofreading mutations in endometrial cancer. J. Natl. Cancer Inst..

[31] McConechy M.K., Talhouk A., Leung S., Chiu D., Yang W., Senz J., Reha-Krantz L.J., Lee C.H., Huntsman D.G., Gilks C.B. (2016). Endometrial Carcinomas with POLE Exonuclease Domain Mutations Have a Favorable Prognosis. Clin. Cancer Res..

[32] Gargiulo P., Della Pepa C., Berardi S., Califano D., Scala S., Buonaguro L., Ciliberto G., Brauchli P., Pignata S. (2016). Tumor genotype and immune microenvironment in POLE-ultramutated and MSI-hypermutated Endometrial Cancers: New candidates for checkpoint blockade immunotherapy?. Cancer Treat. Rev..

[33] Barnum K.J., O’Connell M.J. (2014). Cell cycle regulation by checkpoints. Methods Mol. Biol..

[34] Lin Z.P., Zhu Y.L., Ratner E.S. (2018). Targeting Cyclin-Dependent Kinases for Treatment of Gynecologic Cancers. Front. Oncol..

[35] Chohan T.A., Qayyum A., Rehman K., Tariq M., Akash M.S.H. (2018). An insight into the emerging role of cyclin-dependent kinase inhibitors as potential therapeutic agents for the treatment of advanced cancers. Biomed. Pharmacother.

[36] Lim S., Kaldis P. (2013). Cdks, cyclins and CKIs: Roles beyond cell cycle regulation. Development.

[37] Malumbres M., Harlow E., Hunt T., Hunter T., Lahti J.M., Manning G., Morgan D.O., Tsai L.H., Wolgemuth D.J. (2009). Cyclin-dependent kinases: A family portrait. Nat. Cell Biol..

[38] Harbour J.W., Luo R.X., Dei Santi A., Postigo A.A., Dean D.C. (1999). Cdk phosphorylation triggers sequential intramolecular interactions that progressively block Rb functions as cells move through G1. Cell.

[39] Malumbres M. (2014). Cyclin-dependent kinases. Genome Biol..

[40] Schneider E., Kartarius S., Schuster N., Montenarh M. (2002). The cyclin H/cdk7/Mat1 kinase activity is regulated by CK2 phosphorylation of cyclin H. Oncogene.

[41] Romano D. Comparison of preclinical data across CDK 4-6 inhibitors. Proceedings of the ESMO Symposium on Signalling Pathways in Cancer 2018.

[42] Parry D., Guzi T., Shanahan F., Davis N., Prabhavalkar D., Wiswell D., Seghezzi W., Paruch K., Dwyer M.P., Doll R. (2010). Dinaciclib (SCH 727965), a novel and potent cyclin-dependent kinase inhibitor. Mol. Cancer Ther..

[43] Carey J.P.W., Karakas C., Bui T., Chen X., Vijayaraghavan S., Zhao Y., Wang J., Mikule K., Litton J.K., Hunt K.K. (2018). Synthetic Lethality of PARP Inhibitors in Combination with MYC Blockade Is Independent of BRCA Status in Triple-Negative Breast Cancer. Cancer Res..

[44] Christian B.A., Grever M.R., Byrd J.C., Lin T.S. (2007). Flavopiridol in the treatment of chronic lymphocytic leukemia. Curr. Opin. Oncol..

[45] Phelps M.A., Lin T.S., Johnson A.J., Hurh E., Rozewski D.M., Farley K.L., Wu D., Blum K.A., Fischer B., Mitchell S.M. (2009). Clinical response and pharmacokinetics from a phase 1 study of an active dosing schedule of flavopiridol in relapsed chronic lymphocytic leukemia. Blood.

[46] Blum W., Phelps M.A., Klisovic R.B., Rozewski D.M., Ni W., Albanese K.A., Rovin B., Kefauver C., Devine S.M., Lucas D.M. (2010). Phase I clinical and pharmacokinetic study of a novel schedule of flavopiridol in relapsed or refractory acute leukemias. Haematologica.

[47] Ghia P., Scarfò L., Perez S., Pathiraja K., Derosier M., Small K., McCrary Sisk C., Patton N. (2017). Efficacy and safety of dinaciclib vs. ofatumumab in patients with relapsed/refractory chronic lymphocytic leukemia. Blood.

[48] Spring L.M., Zangardi M.L., Moy B., Bardia A. (2017). Clinical Management of Potential Toxicities and Drug Interactions Related to Cyclin-Dependent Kinase 4/6 Inhibitors in Breast Cancer: Practical Considerations and Recommendations. Oncologist.

[49] Dickler M.N., Tolaney S.M., Rugo H.S., Cortés J., Diéras V., Patt D., Wildiers H., Hudis C.A., O’Shaughnessy J., Zamora E. (2017). MONARCH 1, A Phase II Study of Abemaciclib, a CDK4 and CDK6 Inhibitor, as a Single Agent, in Patients with Refractory HR. Clin. Cancer Res..

[50] O’Leary B., Finn R.S., Turner N.C. (2016). Treating cancer with selective CDK4/6 inhibitors. Nat.Rev.Clin. Oncol..

[51] DeCaprio J.A., Ludlow J.W., Lynch D., Furukawa Y., Griffin J., Piwnica-Worms H., Huang C.M., Livingston D.M. (1989). The product of the retinoblastoma susceptibility gene has properties of a cell cycle regulatory element. Cell.

[52] Barnes D.M., Gillett C.E. (1998). Cyclin D1 in breast cancer. Breast Cancer Res.Treat..

[53] Ertel A., Dean J.L., Rui H., Liu C., Witkiewicz A.K., Knudsen K.E., Knudsen E.S. (2010). RB-pathway disruption in breast cancer: Differential association with disease subtypes, disease-specific prognosis and therapeutic response. Cell Cycle.

[54] Sorlie T., Tibshirani R., Parker J., Hastie T., Marron J.S., Nobel A., Deng S., Johnsen H., Pesich R., Geisler S. (2003). Repeated observation of breast tumor subtypes in independent gene expression data sets. Proc. Natl Acad Sci USA.

[55] Asghar U.S., Barr A.R., Cutts R., Beaney M., Babina I., Sampath D., Giltnane J., Lacap J.A., Crocker L., Young A. (2017). Single-Cell Dynamics Determines Response to CDK4/6 Inhibition in Triple-Negative Breast Cancer. Clin. Cancer Res..

[56] Finn R.S., Dering J., Conklin D., Kalous O., Cohen D.J., Desai A.J., Ginther C., Atefi M., Chen I., Fowst C. (2009). PD 0332991, a selective cyclin D kinase 4/6 inhibitor, preferentiallyinhibits proliferation of luminal estrogen receptor-positive humanbreast cancer cell lines in vitro. Breast Cancer Res..

[57] O’Brien N.A., Tomaso E.D., Ayala R., Tong L. (2014). Abstract 4756: In vivo efficacy of combined targeting of CDK 4/6, ER and PI3K signaling in ER+ breast cancer. Cancer Res..

[58] Thangavel C., Dean J.L., Ertel A., Knudsen K.E., Aldaz C.M., Witkiewicz A.K., Clarke R., Knudsen E.S. (2011). Therapeutically activating RB: Reestablishing cell cycle control in endocrine therapy-resistant breast cancer. Endocr-Relat. Cancer.

[59] Cardoso F., Senkus E., Costa A., Papadopoulos E., Aapro M., André F., Harbeck N., Aguilar Lopez B., Barrios C.H., Bergh J. (2018). 4th ESO-ESMO International Consensus Guidelines for Advanced Breast Cancer (ABC 4)†. Ann. Oncol..

[60] De Luca A., Maiello M.R., D’Alessio A., Frezzetti D., Gallo M., Carotenuto M., Normanno N. (2018). Pharmacokinetic drug evaluation of palbociclib for the treatment of breast cancer. Expert. Opin. Drug Metab. Toxicol..

[61] Toogood P.L., Harvey P.J., Repine J.T., Sheehan D.J., VanderWel S.N., Zhou H., Keller P.R., McNamara D.J., Sherry D., Zhu T. (2005). Discovery of a potent and selective inhibitor of cyclin-dependent kinase 4/6. J. Med. Chem..

[62] Finn R.S., Martin M., Rugo H.S., Jones S., Im S.A., Gelmon K., Harbeck N., Lipatov O.N., Walshe J.M., Moulder S. (2016). Palbociclib and Letrozole in Advanced Breast Cancer. N. Engl. J. Med..

[63] O’Leary B., Cutts R.J., Liu Y., Hrebien S., Huang X., Fenwick K., André F., Loibl S., Loi S., Garcia-Murillas I. (2018). The Genetic Landscape and Clonal Evolution of Breast Cancer Resistance to Palbociclib plus Fulvestrant in the PALOMA-3 Trial. Cancer Discov..

[64] Corona S.P., Generali D. (2018). Abemaciclib: A CDK4/6 inhibitor for the treatment of HR+/HER2- advanced breast cancer. Drug Des. Devel. Ther..

[65] Gong X., Litchfield L.M., Webster Y., Chio L.C., Wong S.S., Stewart T.R., Dowless M., Dempsey J., Zeng Y., Torres R. (2017). Genomic Aberrations that Activate D-type Cyclins Are Associated with Enhanced Sensitivity to the CDK4 and CDK6 Inhibitor Abemaciclib. Cancer Cell.

[66] Sledge G.W., Toi M., Neven P., Sohn J., Inoue K., Pivot X., Burdaeva O., Okera M., Masuda N., Kaufman P.A. (2017). MONARCH 2: Abemaciclib in Combination With Fulvestrant in Women With HR+/HER2- Advanced Breast Cancer Who Had Progressed While Receiving Endocrine Therapy. J. Clin. Oncol..

[67] Goetz M.P., Toi M., Campone M., Sohn J., Paluch-Shimon S., Huober J., Park I.H., Trédan O., Chen S.C., Manso L. (2017). MONARCH 3: Abemaciclib As Initial Therapy for Advanced Breast Cancer. J. Clin. Oncol..

[68] Hortobagyi G.N. (2018). Ribociclib for the first-line treatment of advanced hormone receptor-positive breast cancer: A review of subgroup analyses from the MONALEESA-2 trial. Breast Cancer Res..

[69] Hortobagyi G.N., Stemmer S.M., Burris H.A., Yap Y.S., Sonke G.S., Paluch-Shimon S., Campone M., Petrakova K., Blackwell K.L., Winer E.P. (2018). Updated results from MONALEESA-2, a phase III trial of first-line ribociclib plus letrozole versus placebo plus letrozole in hormone receptor-positive, HER2-negative advanced breast cancer. Ann. Oncol..

[70] Fang H., Huang D., Yang F., Guan X. (2018). Potential biomarkers of CDK4/6 inhibitors in hormone receptor-positive advanced breast cancer. Breast Cancer Res. Treat..

[71] DeMichele A., Clark A.S., Tan K.S., Heitjan D.F., Gramlich K., Gallagher M., Lal P., Feldman M., Zhang P., Colameco C. (2015). CDK 4/6 inhibitor palbociclib (PD0332991) in Rb+ advanced breast cancer: Phase II activity, safety, and predictive biomarker assessment. Clin. Cancer Res..

[72] Herrera-Abreu M.T., Palafox M., Asghar U., Rivas M.A., Cutts R.J., Garcia-Murillas I., Pearson A., Guzman M., Rodriguez O., Grueso J. (2016). Early Adaptation and Acquired Resistance to CDK4/6 Inhibition in Estrogen Receptor-Positive Breast Cancer. Cancer Res..

[73] Wander S.A., Cohen O., Johnson G.N., Kim D., Luo F., Mao P., Nayar U., Helvie K., Marini L., Freeman S. (2018). Whole exome sequencing (WES) in hormone-receptor positive (HR+) metastatic breast cancer (MBC) to identify mediators of resistance to cyclin-dependent kinase 4/6 inhibitors (CDK4/6i). J. Clin. Oncol..

[74] Tsuda H., Yamamoto K., Inoue T., Uchiyama I., Umesaki N. (2000). The role of p16-cyclin d/CDK-pRb pathway in the tumorigenesis of endometrioid-type endometrial carcinoma. Br. J. Cancer.

[75] Santala S., Talvensaari-Mattila A., Soini Y., Honkavuori-Toivola M., Santala M. (2014). High expression of cyclin A is associated with poor prognosis in endometrial endometrioid adenocarcinoma. Tumour Biol..

[76] Tanaka T., Terai Y., Ashihara K., Fujiwara S., Tanaka Y., Sasaki H., Tsunetoh S., Ohmichi M. (2017). The efficacy of the cyclin-dependent kinase 4/6 inhibitor in endometrial cancer. PLoS ONE.

[77] Dosil M.A., Mirantes C., Eritja N., Felip I., Navaridas R., Gatius S., Santacana M., Colàs E., Moiola C., Schoenenberger J.A. (2017). Palbociclib has antitumour effects on Pten-deficient endometrial neoplasias. J. Pathol..

[78] Huang K.T., Pavlides S.C., Lecanda J., Blank S.V., Mittal K.R., Gold L.I. (2012). Estrogen and progesterone regulate p27kip1 levels via the ubiquitin-proteasome system: Pathogenic and therapeutic implications for endometrial cancer. PLoS ONE.

[79] Butt A.J., McNeil C.M., Musgrove E.A., Sutherland R.L. (2005). Downstream targets of growth factor and oestrogen signalling and endocrine resistance: The potential roles of c-Myc, cyclin D1 and cyclin E. Endocr. Relat. Cancer.

[80] Altucci L., Addeo R., Cicatiello L., Germano D., Pacilio C., Battista T., Cancemi M., Petrizzi V.B., Bresciani F., Weisz A. (1997). Estrogen induces early and timed activation of cyclin-dependent kinases 4, 5, and 6 and increases cyclin messenger ribonucleic acid expression in rat uterus. Endocrinology.

[81] Hamilton C.A., Cheung M.K., Osann K., Chen L., Teng N.N., Longacre T.A., Powell M.A., Hendrickson M.R., Kapp D.S., Chan J.K. (2006). Uterine papillary serous and clear cell carcinomas predict for poorer survival compared to grade 3 endometrioid corpus cancers. Br. J. Cancer.

[82] Milde-Langosch K., Bamberger A.M., Goemann C., Rössing E., Rieck G., Kelp B., Löning T. (2001). Expression of cell-cycle regulatory proteins in endometrial carcinomas: Correlations with hormone receptor status and clinicopathologic parameters. J. Cancer Res. Clin. Oncol..

[83] Cocco E., Lopez S., Black J., Bellone S., Bonazzoli E., Predolini F., Ferrari F., Schwab C.L., Menderes G., Zammataro L. (2016). Dual CCNE1/PIK3CA targeting is synergistic in CCNE1-amplified/PIK3CA-mutated uterine serous carcinomas in vitro and in vivo. Br. J. Cancer.

[84] Flaherty K.T., Lorusso P.M., Demichele A., Abramson V.G., Courtney R., Randolph S.S., Shaik M.N., Wilner K.D., O’Dwyer P.J., Schwartz G.K. (2012). Phase I, dose-escalation trial of the oral cyclin-dependent kinase 4/6 inhibitor PD 0332991, administered using a 21-day schedule in patients with advanced cancer. Clin. Cancer Res..

[85] Infante J.R., Cassier P.A., Gerecitano J.F., Witteveen P.O., Chugh R., Ribrag V., Chakraborty A., Matano A., Dobson J.R., Crystal A.S. (2016). A Phase I Study of the Cyclin-Dependent Kinase 4/6 Inhibitor Ribociclib (LEE011) in Patients with Advanced Solid Tumors and Lymphomas. Clin. Cancer Res..

[86] Patnaik A., Rosen L.S., Tolaney S.M., Tolcher A.W., Goldman J.W., Gandhi L., Papadopoulos K.P., Beeram M., Rasco D.W., Hilton J.F. (2016). Efficacy and Safety of Abemaciclib, an Inhibitor of CDK4 and CDK6, for Patients with Breast Cancer, Non-Small Cell Lung Cancer, and Other Solid Tumors. Cancer Discov..

[87] Benson C., White J., De Bono J., O’Donnell A., Raynaud F., Cruickshank C., McGrath H., Walton M., Workman P., Kaye S. (2007). A phase I trial of the selective oral cyclin-dependent kinase inhibitor seliciclib (CYC202; R-Roscovitine), administered twice daily for 7 days every 21 days. Br. J. Cancer.

[88] Tolaney S.M., Frederick H.J., M C.J. (2017). Phase 1 study of sapacitabine and seliciclib in patients with advanced solid tumors. J. Clin. Oncol..

[89] Miller T.W., Balko J.M., Fox E.M., Ghazoui Z., Dunbier A., Anderson H., Dowsett M., Jiang A., Smith R.A., Maira S.M. (2011). ERα-dependent E2F transcription can mediate resistance to estrogen deprivation in human breast cancer. Cancer Discov..

[90] Vora S.R., Juric D., Kim N., Mino-Kenudson M., Huynh T., Costa C., Lockerman E.L., Pollack S.F., Liu M., Li X. (2014). CDK 4/6 inhibitors sensitize PIK3CA mutant breast cancer to PI3K inhibitors. Cancer Cell.

[91] Teh J.L.F., Aplin A.E. (2019). Arrested Developments: CDK4/6 Inhibitor Resistance and Alterations in the Tumor Immune Microenvironment. Clin. Cancer Res..

[92] Ameratunga M., Kipps E., Okines A.F.C., Lopez J.S. (2019). To Cycle or Fight-CDK4/6 Inhibitors at the Crossroads of Anticancer Immunity. Clin. Cancer Res..

[93] Karnezis A.N., Leung S., Magrill J., McConechy M.K., Yang W., Chow C., Kobel M., Lee C.H., Huntsman D.G., Talhouk A. (2017). Evaluation of endometrial carcinoma prognostic immunohistochemistry markers in the context of molecular classification. J. Pathol. Clin. Res..

[94] Zhang Y., Zhao D., Gong C., Zhang F., He J., Zhang W., Zhao Y., Sun J. (2015). Prognostic role of hormone receptors in endometrial cancer: A systematic review and meta-analysis. World J. Surg Oncol..

[95] Holst F., Hoivik E.A., Gibson W.J., Taylor-Weiner A., Schumacher S.E., Asmann Y.W., Grossmann P., Trovik J., Necela B.M., Thompson E.A. (2016). Recurrent hormone-binding domain truncated ESR1 amplifications in primary endometrial cancers suggest their implication in hormone independent growth. Sci. Rep..

[96] Bogliolo S., Cassani C., Gardella B., Musacchi V., Babilonti L., Venturini P.L., Ferrero S., Spinillo A. (2015). Current opinion on bevacizumab on endometrial cancer treatment. Expert. Opin. Biol. Ther..

